# Lead Exposure in Bald Eagles from Big Game Hunting, the Continental Implications and Successful Mitigation Efforts

**DOI:** 10.1371/journal.pone.0051978

**Published:** 2012-12-19

**Authors:** Bryan Bedrosian, Derek Craighead, Ross Crandall

**Affiliations:** Craighead Beringia South, Kelly, Wyoming, United States of America; USGS National Wildlife Health Center, United States of America

## Abstract

Studies suggest hunter discarded viscera of big game animals (i.e., offal) is a source of lead available to scavengers. We investigated the incidence of lead exposure in bald eagles in Wyoming during the big game hunting season, the influx of eagles into our study area during the hunt, the geographic origins of eagles exposed to lead, and the efficacy of using non-lead rifle ammunition to reduce lead in eagles. We tested 81 blood samples from bald eagles before, during and after the big game hunting seasons in 2005–2010, excluding 2008, and found eagles had significantly higher lead levels during the hunt. We found 24% of eagles tested had levels indicating at least clinical exposure (>60 ug/dL) during the hunt while no birds did during the non-hunting seasons. We performed driving surveys from 2009–2010 to measure eagle abundance and found evidence to suggest that eagles are attracted to the study area during the hunt. We fitted 10 eagles with satellite transmitters captured during the hunt and all migrated south after the cessation of the hunt. One returned to our study area while the remaining nine traveled north to summer/breed in Canada. The following fall, 80% returned to our study area for the hunting season, indicating that offal provides a seasonal attractant for eagles. We fitted three local breeding eagles with satellite transmitters and none left their breeding territories to feed on offal during the hunt, indicating that lead ingestion may be affecting migrants to a greater degree. During the 2009 and 2010 hunting seasons we provided non-lead rifle ammunition to local hunters and recorded that 24% and 31% of successful hunters used non-lead ammunition, respectively. We found the use of non-lead ammunition significantly reduced lead exposure in eagles, suggesting this is a viable solution to reduce lead exposure in eagles.

## Introduction

Lead exposure in terrestrial birds has received much attention in recent years both in North America and Europe (for reviews, see [1], [2]). There are studies that describe lead fragmentation of rifle bullets in the carcasses and offal (i.e., gut piles) of ground squirrels (*Spermophilus richardsonii*), prairie dogs (*Cynomys ludovicianus*), deer (*Odocioleus* spp.), roe deer (*Capreolus capreolus*), elk, fallow deer, and red deer (*Cervus spp)*, [3]–[7], and all make the argument that these lead fragments pose a hazard to scavenging species. Several studies have focused on lead ingestion of rifle bullet fragments in the critically endangered California Condors (*Gymnogyps californianus*) because of the large percentage of free-flying condors that have symptoms of and/or have died from lead poisoning (e.g., [8]–[10]). There is isotopic evidence that the majority of lead ingested by condors originates from spent rifle bullets in offal and shot big game un-retrieved by hunters [8], thus substantiating the earlier suppositions that avian scavengers can incur lead poisoning from big game hunting practices [11]–[13], [7]. Similarly, Common Ravens (*Corvus corax*) and Turkey Vultures (*Cathartes aura*) have significantly higher blood lead levels during big game hunting seasons than non-hunting periods [6], [14], [15] offering further evidence that lead ingestion from offal poses a risk to all avian scavengers.

There have been several studies on lead exposure in eagles across North America. The incidence of lead ingestion in both bald eagles (*Haliaeetus leucocephalus*) and golden eagles (*Aquila chrysaetos*) did not change after the ban of lead shot for waterfowl hunting [16], suggesting offal as an alternate source of lead exposure. Two studies [13], [17] both found high incidence of lead poisoning in eagles and found that the times and areas of high exposure were not correlated to waterfowl hunting for both the western US and the Great Plains. Both studies suggested that big game hunting may be a significant source of dietary lead exposure for eagles. A spatial-temporal association with lead exposure and big game hunting seasons has been found for both bald and golden eagles in California, the Pacific Northwest, and the Midwest, [12], [18], [19], respectively. Most recently, bald eagle admission to rehabilitation facilities in the Midwest have indicated a positive relationship of lead exposure to the rifle hunting season and zones from 1996–2009 as well as a correlation between lead and copper exposure in the eagles, further suggesting big-game hunting ammunition as the source [20]. While all of these studies on lead exposure in eagles support the hypothesis that big game hunting is a significant source of lead exposure for eagles, the data are correlative. No experimental data exist to determine if reducing the amount of lead ammunition used reduces lead exposure in eagles.

Few studies have examined blood lead levels of live, free-ranging eagles. Lead exposure has been found in 36% of 162 golden eagles sampled year-round [12], but the majority of exposure occurred during the deer hunting season. Eighty-six percent of migrant bald eagles in Montana (n = 37) were found with elevated lead levels [21], but there was a lower exposure rate for migrant golden eagles (56%; n = 86). Bald eagles sampled from two different sites in Saskatchewan and Montana during the autumn had 18% and 8% exposure rates [13] (n = 97 and 81, respectively). Golden eagles sampled from recent years in California were found with a 77% lead exposure rate that dropped to 37% following lead rifle ammunition regulation [22]. Most recently, free-ranging bald eagles captured in southwest Montana had significantly higher lead exposure rates in the fall than birds sampled in winter and spring [23]. This study also found eagles sampled in recent years had higher lead loads than eagles sampled after the ban of lead shotgun ammunition for waterfowl hunting, further suggesting lead exposure is likely from rifle ammunition sources.

Recent legislation in California banning the use of non-lead rifle ammunition in the range of the California condor is estimated by the CA Fish and Game Commission to have 80–95% compliance. This appears to be successful in reducing the incidence of lead ingestion by some avian scavengers [22], but not condors [24]. Similarly, a voluntary non-lead bullet program has been in place since 2005 in the Kaibab Plateau of Arizona’s condor range and compliance is estimated at 85% [25] but condor deaths from lead ingestion continues ([26], C. Parish pers. comm.). This lack of response from condor populations is because of the large home ranges of condors, due to the temporal variation in their food sources, and the difficulties in controlling for recreational and predator hunting in field studies [27]. However, this lack of response means that more evidence is needed to convince the general public that non-lead programs can be effective in reducing deaths from lead ingestion in scavenging species such as condors and eagles.

To examine the relationship between lead ammunition and bald eagles, we were interested in addressing the following questions: 1) are bald eagles in the upper Snake River ecosystem being exposed to lead at a higher rate during (or as a result of) the local hunting season 2) can bald eagle blood lead levels be reduced by increasing the amount of non-lead ammunition used by hunters. To address these questions, we sampled blood lead levels of eagles before, during, and after annual big game hunts and nestling bald eagles within this area as a control. We investigated the spatial and temporal exposure risks by satellite tracking eagles and performing regular driving surveys to measure eagle abundance within our study area. To test if lead exposure in bald eagles can be reduced by using non-lead rifle ammunition alternatives, we provided hunters with free or discounted non-lead ammunition (ammunition with copper or gilding metal bullets) and surveyed the proportion of successful hunters using non-lead for two years. Using these data, we modeled the lead levels of bald eagles during this time period to investigate the efficacy of our program.

## Materials and Methods

### Ethics Statement

Animal capture, handling, and sampling protocols were covered under federal and state permits (U.S. Geological Survey bird banding permit #22637; National Park Service scientific collecting permit #GRTE SCI-003; US Dept. of Interior – National Elk Refuge Station #61550, Permit #11-06; Wyoming Game and Fish Department scientific collecting permit #293). The study was approved by the Craighead Beringia South Institutional Animal Care and Use Committee (protocol #CBS04-005-01).

### Study Area

Eagles were studied and captured within the Jackson Hole valley of northwestern Wyoming (43°91′N, 110°40′W). Jackson Hole is an inter-mountain valley (elevation of the valley floor approx. 2300 m) and comprises the headwaters of the Snake River drainage. The valley is composed of mainly public lands including Grand Teton National Park, the National Elk Refuge, and 3 wilderness areas in the surrounding 4 national forests (Bridger-Teton, Targhee, Caribou, and Shoshone). Elk, deer, moose, and bison (*Bison bison*) hunting occurs annually within the valley and most hunters leave offal from their kills in the field (see [6] for a detailed description). Hunting for elk and bison is permitted on the National Elk Refuge and Grand Teton National Park has an elk reduction program. There are roughly 3000 big game animals harvested annually within and directly surrounding the study area [6]. The big game hunting seasons for elk began the second weekend in September and ended the second weekend in December. Little to no recreational or predator hunting occurs outside of the big game hunting season due to the protection afforded by the national park and refuge.

### Capture and Handling

Eagles were captured during and after the hunting seasons using net launchers (CODA Ent., Mesa, AZ and Trapping Innovations, LLC, Kelly, WY). Eagles were baited with road-killed carrion or with existing gut piles opportunistically found in the study area. We captured eagles during the hunting seasons of 2005–07 and 2009–2010 (N = 5, 22, 9, 12, and 7, respectively) and post-hunt in 2007–08 (N = 7 and 4, respectively). Resident eagles were captured pre-hunt in 2010 (N = 7) using a floating noose fish [28] on the Snake River in Jackson Hole. Blood samples from nestlings (N = 8) were taken in cooperation with Grand Teton National Park and A. Harmata (Montana State University) within the Jackson Hole valley during the 2006 nesting season.

Once captured, each eagle received a United States Geological Survey band, was aged, measured, and 2.5 cc of blood was drawn from the brachial vein. Bald Eagles were aged up to six years-old [29] and sex was determined using bill depth and mass [30]. A large portion of the blood sample (2.0 cc) was placed in EDTA storage tubes (Becton, Dickinson, and Company, Kranklin Lakes, NJ) for analysis of blood lead levels (BLL) by inductively coupled plasma mass spectrometry (ICMPS) by the Diagnostic Center for Population and Animal Health (Michigan State University, Lansing, MI). The lower detection level for blood lead levels via ICPMS was 0.1 ug/dL. The remainder of the blood sample was stored in lysis buffer [31] for later DNA analysis. Blood samples from recaptures (n = 2) collected at least three weeks apart were considered independent samples because the lead depuration rate for birds is approximately three weeks [32], [6]. We also confirmed this depuration rate by holding one sub-adult bald eagle with toxic levels for 17 d during which time its’ BLL dropped from 113 ug/dL to below 25 ug/dL. Because of this depuration rate, we did not collect blood samples from mid-December through early-January to prevent ambiguity in the hunting and post-hunting season comparison.

### Non-Lead Ammunition Program

During the hunting season in 2009 we gave away free non-lead ammunition to hunters in Grand Teton National Park and the National Elk Refuge and in 2010 we sold this ammunition at a discount to all hunters in the Jackson Hole region. We purchased Federal® ammunition packed with Barnes® TSX or TTSX bullets, Winchester® E-tip, and Hornady® GMX ammunition from Cabela’s Inc® for our distribution program. We collected survey information from Grand Teton National Park and the National Elk Refuge in all years to determine the number of elk harvested and the number of successful hunters using lead and non-lead ammunition. All successful Park and Refuge elk hunters are required to turn in a hunting survey. Because bison hunters are not required to turn in a hunt survey, we extrapolated the annual percentage of elk hunters using non-lead ammunition to the total bison harvest within the National Elk Refuge (bison hunting is not allowed in Grand Teton National Park).

### Satellite Tracking

During the 2009/10 hunting season we fit 10 eagles, 9 migrant and one local, with 65 g satellite platform terminal transmitters (PTTs; Wildlife Computers) using a backpack style harness with Teflon ribbon and breakaway stitching [33]. During the summer of 2010 we fit 3 additional local breeding eagles to determine their seasonal movements. We reduced the dataset to location accuracy class 3-1 [34] and analyzed the locations in ArcMap 9.3.

### Driving Surveys

**Figure 1:**
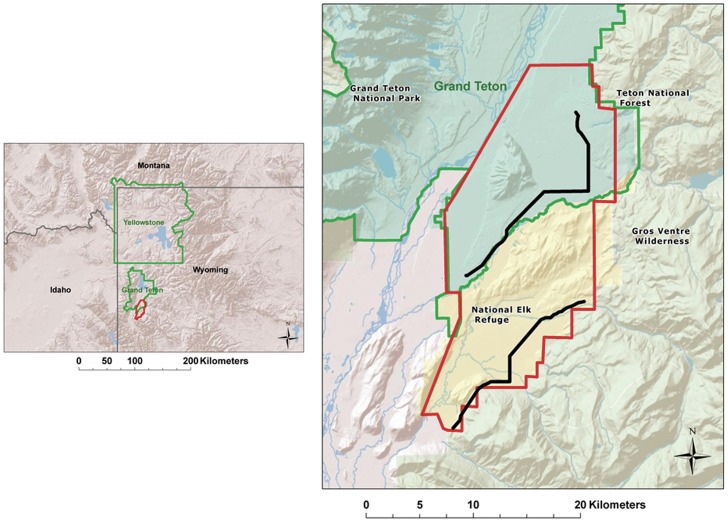
Study area with eagle count transects. Boundary (red) and land ownership of the study area. Eagle count transects outlined in black. The majority of elk harvest in Grand Teton National Park occurs within the study area boundary and the study area includes all hunt zones on the National Elk Refuge.

From August 2009 through March 2011, we performed 126 driving surveys for all corvids and raptors across our study area [35]. Surveys were performed only to detect seasonal abundance shifts within the study area as they relate to the big game hunting season and not density of the species being counted due to several potential biases that exist in raptor road counts [35], such as monitoring only from roads and distribution of elk kills during the hunting season. Weekly surveys were performed seven weeks before and after the hunting season and for the 10 and 11 weeks during each of the hunting seasons in 2009 and 2010, respectively. Surveys were performed bi-weekly during the spring and summer (mid-February through mid-August). We surveyed two different routes (14.5 km and 17.1 km) that bisected our study area on each survey day for a total of 63 repetitions per transect (Figure 1). Routes were chosen to minimize paralleling power lines because of a potential for bias for perching raptors, such as eagles. Driving speed was maintained at 32 km/hr. A team of 2 authors performed the surveys. We alternated starting points to reduce potential time-of-day bias and we recorded species, age, distance to the bird, height of the bird (when first sighted), activity (i.e., perched, flying, feeding, and aggregation) and if it was perched on a power pole.

### Data Analysis

D’Agostino-Pearson normality tests indicated that the blood lead level data were right skewed (P<0.001), so we log transformed the data to achieve a normal distribution (P = 0.378) and used those data in parametric tests. We first tested for differences between age and sex since these variables have influenced lead exposure in other species [36], [6]. We categorized age into adult (>5 yrs.) and sub-adult (0–5 yrs.) and tested for a difference between categories of both age and sex using t-tests. To examine potential differences between hunting and non-hunting seasons, we excluded nestling samples and used a generalized linear mixed model test using blood lead level as the dependent variable and age class, hunt vs non-hunting season, year captured and gender as independent variables with capture date as the random-effects variable. Following the mixed-effects model, we tested for differences between years of any significant factors using an ANVOA. We tested the difference in blood lead levels between the hunting seasons, non-hunting seasons, and nestling lead levels using an ANOVA with post-hoc t-test comparisons. We also tested the proportion of samples during the hunting and non-hunting seasons in lead exposure categories (background, sub-clinical, clinical, and acute exposure) versus expected using a chi-square test.

We were interested in the relative abundance (not density) of eagles within our study area to determine if there was an influx of eagles during the hunting season, so we analyzed the survey data using eagles detected/km. We combined survey data from all non-hunting season surveys (pre and post 2009 and 2010 hunting seasons, N = 42) into one non-hunt abundance sample. We also combined data from both 2009 and 2010 hunts (N = 21) for a hunting season abundance measure. We tested for differences in abundance between the hunting and non-hunting seasons using a Mann-Whitney test because both datasets exhibited non-normal distributions.

We used linear mixed-effects models to examine the relationships between eagle lead levels, harvest, age and sex using capture date as a random effect. The total number of harvested game annually can influence annual blood lead levels of scavenger populations [14], so it is important to adjust for the annual harvest rate when comparing between-year variation in blood lead levels. To test the efficacy of our non-lead program on reducing lead ingestion rates in 2009 and 2010 compared to earlier years, we assessed model fit of mean annual log-transformed lead levels between the total elk and bison harvest and total harvest from hunters using lead ammunition only (discounting the non-lead ammunition harvest). We predicted that if the annual mean population lead level was directly related to the amount of lead ammunition used by hunters (i.e., more lead ammunition = higher lead levels), then the models including the lead ammunition only would have a better fit than the models using total harvest (lead and non-lead harvest combined). We evaluated sex proportion and eagle age structure (as a binary variables; adult and non-adult) to determine if they improved model fit for both harvest models. We used Pearson’s goodness-of-fit coefficients to assess relationship between explanatory variables and blood lead levels. We used Akiake’s information criterion (ΔAIC) [37] to choose top models describing the relationship between our chosen variables and blood lead levels to determine if our non-lead program decreased eagle lead levels (i.e., the lead-only harvest model had a better fit relative to the model using total harvest rate).

## Results

We tested 81 blood samples for blood lead concentrations from 71 free-flying eagles, including two recaptures >3 weeks after initial capture and 8 nestlings (Table 1). Based on the background exposure level criterion of 10 ug/dL [15] and sub-clinical, clinical, and acute levels [38] we found 93% (N = 68) of all non-nestling eagles tested had been exposed to lead. Thirty-three percent of samples (N = 14) exhibited at least clinical lead exposure (>60 ug/dL) and all were sampled during the hunting season. All nestling’s sampled (N = 8) were below 1.0 ug/dL (Table 2) and one nestling had blood lead levels below the detectability threshold.

**Table 1:** Blood lead level (µg/dL) descriptive statistics for bald eagles in Jackson Hole, Wyoming, USA (2005–2007, 2009–10).

	*n*	mean	SE	median	range
Nestling Controls	8	0.29	0.09	0.25	non-detect - 0.8
All Non-Nestlings	73	77.73	13.94	40	2.0–717.9
Non-Hunting Season	18	23.03	3.76	21.4	2.0–53.7
Hunting Season	55	95.63	17.84	55.9	4.3–717.9

**Table 2:** Percentage and number (N) of bald eagle samples in four exposure categories captured between November – January in Jackson Hole, Wyoming, USA from 2005–07, 2009–10.

	Background	Sub-ClinicalExposure	ClinicalExposure	AcuteExposure
	<10 ug/dL	10–59 ug/dL	60–100 ug/dL	>100 ug/dL
Nestlings	100 (8)	0	0	0
All non-nestlings	7 (5)	60 (44)	14 (10)	19 (14)
* Non-Hunting Season*	*22 (4)*	*78 (14)*	*0*	*0*
* Hunting Season*	*2 (1)*	*55 (30)*	*18 (10)*	*25 (14)*

Blood lead levels <10 µg/dL = background, 10–59 µg/dL = exposed, 60–99 µg/dL = clinically affected, and >100 = acute lead exposure (guidelines based on Redig 1984 and Kelly et al. 2011).

**Figure 2:**
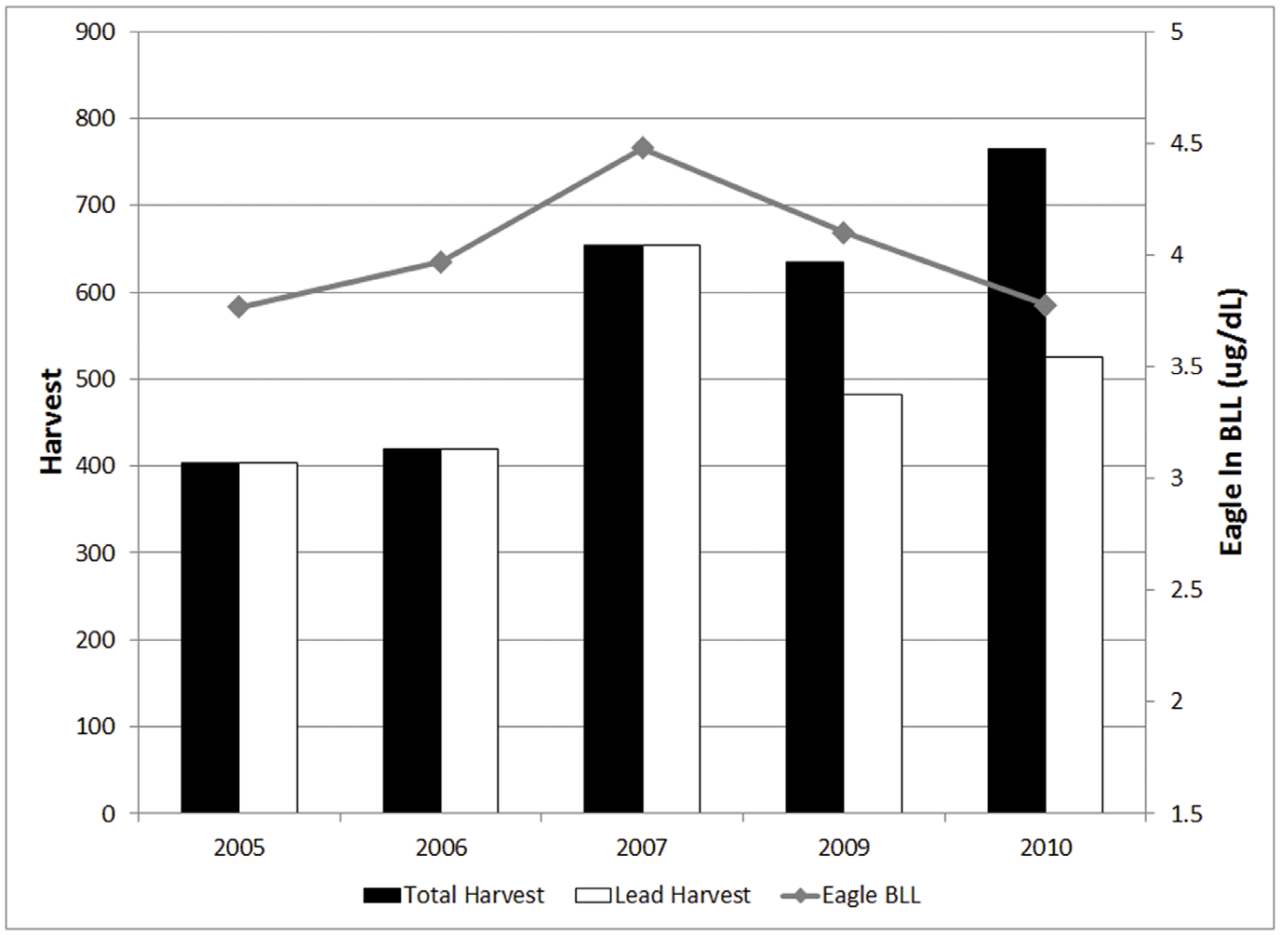
Big game harvest and eagle blood lead levels. Total big game harvest (lead and non-lead) by year (black bars, 2005–2008, 2009–2010) and harvest of big game using only lead-based ammunition (white bars) in Jackson Hole, Wyoming. Log transformed mean seasonal blood lead levels of bald eagles (gray line). Note the x-axis is not time contiguous since eagles were not sampled during the 2008 hunting season.

In 2009, we distributed one free box of non-lead ammunition from 14 different caliber rifles to a total of 194 hunters from Grand Teton National Park (GTNP) and the National Elk Refuge (NER). All successful hunters in GTNP and all hunters on the NER (whether successful or not) are required to turn in hunt surveys. In 2009, 24.5% of successful hunters (n = 331) in GTNP and 34% in the NER (n = 143) used non-lead ammunition for their hunt. Combining both hunt areas, a total of 24% of all successful hunters used non-lead ammunition in 2009. In 2010, we sold non-lead ammunition at a reduced rate to hunters. That year, a total of 31% of hunters in GTNP (n = 340) and 33% of hunters on the NER (n = 221) indicated they used non-lead for their successful harvest. Figure 2.

We found no difference in lead levels by sex (t = −1.56, P = 0.12) or age (t = 1.41; P = 0.16), so we pooled those data for the remainder of the analyses. We found that blood lead levels were higher during the hunting season than outside the hunting season (z = 3.06, P = 0.002) while no other variable tested (age, year captured, and sex) had a significant effect on lead levels (all P>0.3). We found that neither hunting nor non-hunting seasons were significantly different between sampling years (P = 0.373 and 0.396, respectively). Nestlings had lower levels of lead than non-nestlings tested during the non-hunting season (t = 10.68, P<0.001) and eagles sampled during the non-hunting seasons had lower lead levels than those tested during the hunting season (t = −4.65, P<0.001). We found that eagles had lead levels lower than expected during the non-hunting season and higher than expected during the hunt (P<0.001) (Table 2).

We found some evidence to suggest that there was an increase in the abundance of eagles within our study area during the hunting seasons as compared to the non-hunting seasons (W = 1265.5, P = 0.06). From our driving surveys, we found an average of 0.09 eagles/km during the non-hunting seasons (range = 0–0.85 eagles/km; SD = 0.17) and 0.28 eagles/km during the hunting season (range = 0–1.63 eagles/km; SD = 0.43).

**Figure 3:**
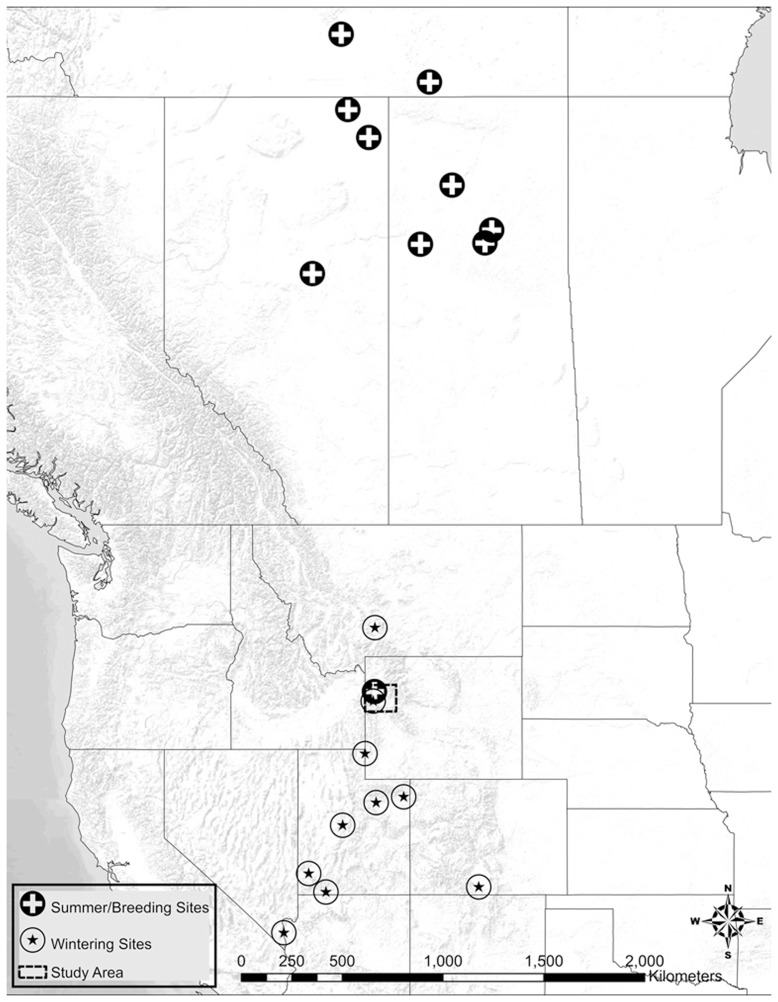
Summer and wintering locations of bald eagles. Winter (2009–2010) and summer locations (2010) of 10 bald eagles fitted with satellite transmitters during the 2009 big game hunting season in Jackson Hole, Wyoming.

**Figure 4:**
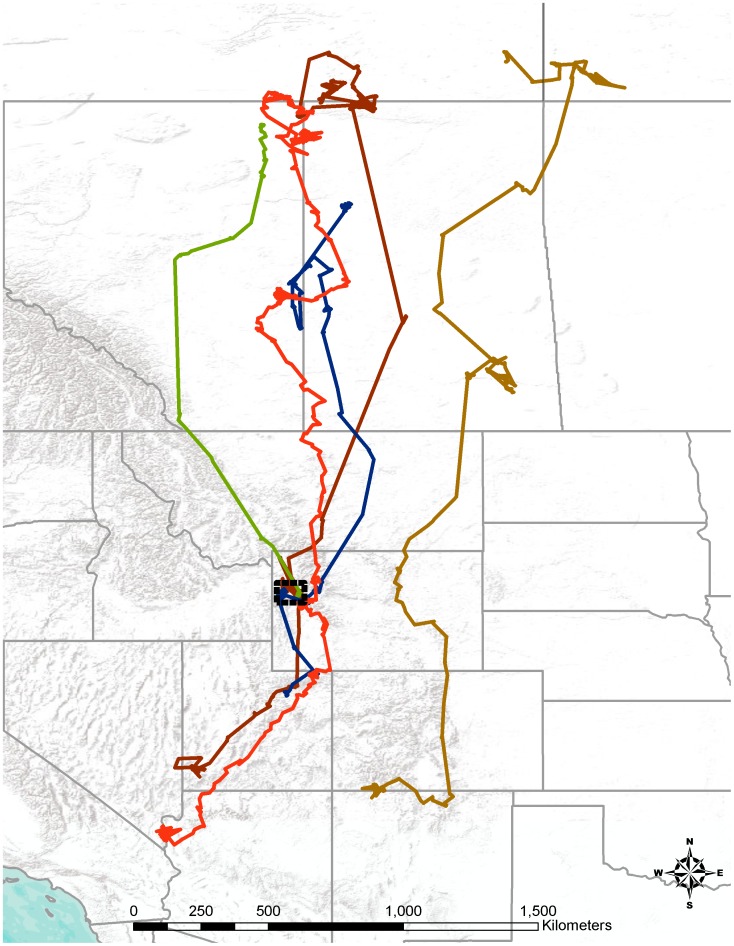
Bald eagle fall migration routes. Fall migratory (August 2010– January 2011) routes of bald eagles captured the previous fall (2009) in Jackson Hole, Wyoming. Four of the five eagles returned to Jackson Hole during the big game hunting season a year after being tagged.

Nine of 10 tagged eagles migrated from the study area after the cessation of the hunting season. The majority (n = 6) wintered in Utah, one each in Montana, Arizona, and Colorado (Figure 2). Almost all of the eagles (n = 9) summered in central Canada while one sub-adult summered in the vicinity of our study area (Figure 3). While in Canada, 4 transmitters stopped working, fell off or the eagle died (unknown since unrecovered) which left 5 functioning transmitters. During the fall migration in 2010, four of the five eagles returned directly to our study area stopping for no more than two days at a time. Once in the study area, they remained for the entire hunting season before continuing their southerly migration [Figure 4]. We fit three adult, breeding eagles within the study area with PTT transmitters during the late summer of 2010 to assess their movement during the hunting season. The average distance from these nests to the nearest hunt zone was 2.3 km (range 1.55–2.83). None of these three individuals left their breeding areas (ca. 55 km^2^) along the Snake River during the hunting season.

Our models suggest that the total amount of elk and bison harvested with non-lead bullets influenced the mean annual lead level (Table 3, Figure 3). The model fit improved when we used the harvest rate with only animals taken using lead ammunition (total harvest – non-lead harvest), as compared to the model using the total harvest (lead harvest+non-lead harvest). There was no relationship between mean lead levels and total harvest across years (P = 0.661, se = .001, coef. <0.001). However, we did find that the mean annual lead level of eagles was related to the level of lead-only harvest (total harvest – non-lead harvest; P = 0.035, se = .001, coef. = 0.002). Neither sex nor age composition helped improve the model fit for either the total harvest or lead-only harvest models.

**Table 3:** Top-ranked models (out of 10 considered) of eagle blood lead levels in relation to harvest with lead-based bullets by age, and sex from 2005–2007, 2009–2010 in Jackson Hole,Wyoming, USA.

Model	Δ AIC	Akaike weight
Lead-Only Harvest^a^	0	0.75
Total Harvest	3.46	0.13
Lead-Only Harvest, Age	4.34	0.09
Lead-Only Harvest, Age, Sex	7.64	0.02
Total Harvest, Age	7.72	0.02

a AIC = 13.48.

## Discussion

Most studies on lead ingestion in scavengers have focused on spatial (i.e., hunting zones) or temporal (i.e., hunting seasons) correlations with lead levels or deaths caused by lead poisoning. Our data suggest a causative relationship between lead rifle ammunition use and lead ingestion in bald eagles. Our study area is devoid of other potential sources of lead ingestion by bald eagles, thereby making it possible to experimentally reduce the potential of lead ingestion by eagles. Because of the protection afforded by the National Elk Refuge and the National Park Service, there is no recreational varmint or predator shooting and no waterfowl or upland game hunting is permitted. Fishing with live bait is also prohibited in the study area, reducing the possibility of the use of lead-based fishing tackle by anglers. Further reducing the possibility of lead exposure through angling is the fact that all sport fish within the study area are in the *Salmonidae* family, which are almost always fished for using non-lead based tackle (i.e., fly-fishing) and virtually no fishing exists during the big-game hunting season due to ice formation on the lakes and rivers within the study area. In our study area, almost all hunters field dress their harvest and leave the gutpile (both thoracic and abdominal organs) at the site of the kill, making it available to scavengers. Thus, by reducing lead ammunition in our study area, we were able to experimentally reduce lead exposure in bald eagles. Our models of lead ingestion in the bald eagle populations of northwestern Wyoming before and after non-lead ammunition was promoted, indicate that lead exposure in eagles can be effectively reduced through the use of non-lead bullets.

Our study area was unique in that for many years it has supported large and concentrated elk and bison herds [39]. Roughly 3,000 elk are harvested within our study area each fall between mid-October and mid-December [6], making a consistent and long-time source of carrion for eagles, not unlike a salmon spawning stream. Our road surveys and satellite tracking data indicate a temporal and spatial shift of eagles into our study area each fall in response to this food source. Bald eagles typically forage and exhibit linear movement patterns associated with rivers and creeks during the summer months within our study area [40]. Our surveys were completed from the roadway with only a portion closely paralleling riparian corridors (Figure 1) so we cannot determine if there was less use of river corridors during the hunting season. However, the data clearly indicate greater eagle use of non-riparian habitats during the big-game hunting season in response to the distribution of offal across the study area.

The local population of breeding bald eagles within our study has been stable (S. Patla, WY Game & Fish Dept., pers. comm.). Fall and winter use areas for the resident eagles were small in comparison to the migrant birds that used the study area in its entirety. The resident use areas were centered along the Snake River near their nest sites where we presume the birds continued to feed primarily on fish and waterfowl. This supposition is also supported by the lack of lead detected in local, nestling bald eagles (Table 2). The big game hunting season in our study area does not overlap the nesting period for bald eagles so the resident adults are likely foraging on food sources not contaminated with lead, such as fish, and feeding the nestlings those prey. It remains unknown if adults mobilize lead stored in bone during egg production since lead can replace calcium reserves in bone [41]. Since blood lead levels only reflect ingestion within the previous two weeks [6] and the eaglets were tested well after hatching, our results cannot help elucidate that theory but do indicate that breeding adults are foraging on prey with little to no lead contamination.

In our study, most satellite tracked migrants returned to central Canada for the spring and summer and returned the following year to Jackson Hole during the hunting season (Figure 4). While we have no information about the health of northern breeding populations, it is germane that lead and its toxic effects are cumulative from year to year [42].

While there is no consensus on background or lethal concentrations of lead for most raptors, most researchers consider a blood concentration >100 ug/dL to indicate acute poisoning. We measured a blood lead concentration of 717 ug/dL in an eagle that subsequently migrated south to Colorado and then north to Northwest Territories, Canada. While several eagles have been found dead with confirmed lead poisoning (liver lead concentrations >6 ppm ww) during the hunting season within our study area (S. Patla pers. comm.), it appears there is large variation among individuals in the amount of lead that can be tolerated. We did not detect any clinical signs of lead toxicity in captured eagles. Eagles were only handled for a short amount of time during a period of induced stress due to capture and handling so clinical signs of lead intoxication may have been overlooked. Our tracking data coupled with high lead burdens indicates more studies need to be conducted on lethal levels and lead burdens that can be tolerated by this species. Cumulative effects on longevity, behaviors, and breeding success remain unknown and may be a significant source of mortality.

Recently, there have been studies linking the lead isotopic ratios in feathers of exposed condors with ammunition sources [8], [43]. Such studies would likely be relevant for eagles to further elucidate the direct link between lead-based rifle ammunition and lead ingestion in scavengers, such as bald eagles. In addition to linking lead-based rifle ammunition to exposure in eagles, several recent studies have raised concern for human health implications arising from lead-based rifle bullets [44], [45]. Fragments from lead-based rifle bullets are not only left in the gutpiles of field dressed big-game, but are also present in packaged meat [44] and may prove useful for investigating this potential source of human dietary lead exposure.

Unlike many environmental problems there is a straightforward and easy solution to toxic lead exposure in wildlife from rifle ammunition: non-lead alternative ammunition. In our study many hunters quickly made this transition voluntarily, 24% and 31% the first and second year, respectively and this resulted in immediate, measurably lower lead levels of eagles. This was accomplished with modest public awareness and by making non-lead ammunition more readily available. There remains a majority of hunters who have not changed and for whom additional and persuasive education and positive incentives will be necessary. In Arizona, a similar program with state support and funding to offer free non-lead ammunition to all hunters obtained an annual average of 85% voluntary compliance [25]. For a state or national program to succeed it will take a concerted and coordinated effort of state and federal wildlife and natural resource agencies and other interested organizations to educate hunters about the superior ballistic performance and environmental benefits of non-lead rifle ammunition. Without proper education, support for and compliance of non-lead regulations will fall short of the thresholds needed to effectively remove this source of lead contamination from wildlife and wildlands.
